# Dysgraphia detection through machine learning

**DOI:** 10.1038/s41598-020-78611-9

**Published:** 2020-12-09

**Authors:** Peter Drotár, Marek Dobeš

**Affiliations:** 1 Department of Computers and Informatics, Technical University of Košice, 04001 Košice, Slovakia; 2 Centre for Social and Psychological Sciences, Slovak Academy of Sciences, 04001 Košice, Slovakia

**Keywords:** Preclinical research, Electrical and electronic engineering

## Abstract

Dysgraphia, a disorder affecting the written expression of symbols and words, negatively impacts the academic results of pupils as well as their overall well-being. The use of automated procedures can make dysgraphia testing available to larger populations, thereby facilitating early intervention for those who need it. In this paper, we employed a machine learning approach to identify handwriting deteriorated by dysgraphia. To achieve this goal, we collected a new handwriting dataset consisting of several handwriting tasks and extracted a broad range of features to capture different aspects of handwriting. These were fed to a machine learning algorithm to predict whether handwriting is affected by dysgraphia. We compared several machine learning algorithms and discovered that the best results were achieved by the adaptive boosting (AdaBoost) algorithm. The results show that machine learning can be used to detect dysgraphia with almost 80% accuracy, even when dealing with a heterogeneous set of subjects differing in age, sex and handedness.

## Introduction

Dysgraphia is a disorder affecting the written expression of symbols and words. In contemporary culture, we still heavily depend on our ability to communicate using written language; therefore, dysgraphia can be a serious problem. In addition, dysgraphia in a school setting can affect the child’s normal development and self-esteem, as well as academic achievements^1,2^. Early diagnosis enables children to seek help and improve their writing sooner and helps teachers adapt their teaching style after properly diagnosing a source of learning difficulty in a child^3^.

Dysgraphia is often associated with other disorders such as dyslexia. On a neurophysiological level, these disorders seem to share similar brain areas^4^. Dysgraphia also shares similarities to developmental coordination disorder^5,6^ and more generalized oral and written language learning disability^7^. Dysgraphia is not a homogeneous construct^8^ and may be represented by different handwriting features^9^. Acquired dysgraphia is usually connected to an injury or illness affecting areas of the brain and is less common^10^. Specific alternations of handwriting frequently occur in Parkinson’s disease patients^11,12^, giving rise to so-called Parkinson’s disease dysgraphia. In this study, we focus on developmental dysgraphia that starts for no obvious reason and is present from an early age.

What does normal development of handwriting look like? Scribbling is an equivalent of writing in young children. A child starts to explore the possibilities of writing gradually in stages, expanding her repertoire of strokes and shapes. After writing is taught in elementary school, writing speed increases, and writing competency reaches adult levels at approximately fifteen years of age^13^. Studies suggest that dysgraphia and related disorders may manifest differently in different age groups^14^. The authors of the recent study^15^ state that at younger ages, handwriting automaticity accounts for as much as 67% of the variance in text quality^15^, while at the middle school level, handwriting automaticity accounts for 16% of the variance in text quality^7,15^.

There have been several attempts to diagnose dysgraphia using machine learning. By using tablets, various handwriting features can be measured and analysed. Features to be extracted, e.g., speed of writing, stops and lifting of a pen, are inspired by neuropsychological and neurological research on dysgraphia^16,17^. Compared with standard clinical testing, which relies mostly on static features such as text shape or writing density and time needed to complete tasks^9^, digitized testing adds features that could not have been measured before, such as pressure, handwriting speed, acceleration and in-air movement. In their work, Asselborn et al.^9^ identify four types of features—static, kinematic, pressure and tilt. Similarly, Mekyska et al.^18^ use kinematic, non-linear dynamic and other features. Rosenbloom et al.^19^ use temporal and product quality features.

Most frequently, three machine learning approaches are used to identify dysgraphia. Asselborn et al.^9^ substitute random forests for traditional BHK testing. Mekyska et al.^18^ use random forests to detect dysgraphia in 8- to 9-year-olds. Another model by Rosenbloom and Dror^19^ uses linear support vector machines (SVMs) to identify dysgraphia in children. Sihwi et al.^20^ also use SVM to identify dysgraphia. However, in their study, children were asked to write directly on a smartphone screen, which creates a setting different from that of usual writing. Neural networks (NNs) are another tool used for dysgraphia identification. Samodro and Sihwi^21^ use simple NNs with 6 hidden neurons. Kariyawasham et al.^22^ use deep learning to screen for dysgraphia. In addition, Asselborn et al.^9^ use K-means clustering with PCA to identify dysgraphia, while others experiment with augmented features used for analysis—for example, Zvoncak et al.^23^ use fractional order derivative features and features based on the tuneable Q-factor wavelet transform^24^.

Machine learning methods are also used to diagnose other learning disabilities, such as dyslexia^25,26^.

In this study, a template for the acquisition of handwriting data was proposed and used as a source for the automated diagnosis of dysgraphia. A number of previously known and new features were extracted and employed to train the machine learning model to identify handwriting affected by dysgraphia. The results show that even in a heterogeneous dataset, a predictive model is able to identify subjects with dysgraphia.

The rest of the paper is organized as follows. In the next section, we provide the details of data acquisition and the obtained dataset. Then, we outline the methods used for preliminary analysis and machine learning algorithms for classification. Finally, in the last section, we present and discuss the experimental results.

## Methods

### Participants and data collection

A total of 120 schoolchildren participated in data collection. Their ages and sex distribution are outlined in Table 1. The dominant hand was the left hand for 16 children and the right hand for the remaining children. The distribution of sex did not have equal probabilities (one-sample binomial distribution test with significance 0.000). Age distribution was normal (one-sample Kolmogorov–Smirnov test with significance 0.055). The t-test concerning age differences between groups of children with and without dysgraphia was not significant. The mean age was not significantly different between the two groups.

**Table 1:** Distribution of sex and age in the dataset.

Age	Boys	Girls	Dysgraphia	Normally developing
8	5	3	4	6
9	7	3	4	5
10	10	4	8	7
11	8	4	5	6
12	15	7	6	16
13	12	6	10	8
14	10	7	9	8
15	13	6	11	7
all	80	40	57	63

Data from children with dysgraphia were collected by trained professionals at the Centre for Special-Needs Education in 2018 and 2019 as part of a standard assessment. Data from children without dysgraphia were collected by trained professionals at their elementary school. This study was undertaken under research grant APVV-16-0211 approved by the Ethical Commission of the University of Pavol Jozef Šafárik in Košice. All research was performed in accordance with relevant guidelines and regulations. Informed consent from a parent and/or legal guardian of each child was obtained. Data are available in public repository (https://github.com/peet292929/Dysgraphia-detection-through-machine-learning) or upon requests from authors. During data acquisition, the subject was in a separate room (not in a classroom). The template used is presented in the Supplementary information file and consisted of writing the letter “l” at normal and fast speeds, writing the syllable “le” at normal and fast speeds, writing the simple word “leto” (summer), writing the pseudoword “lamoken”, writing the difficult word “hračkárstvo” (toy-shop), and writing the sentence “V lete bude teplo a sucho” (The weather in summer is hot and dry).

Subjects with any hand injury or physical indisposition to write were excluded. All subjects had normal or corrected-to-normal vision. Inclusion required diagnosed dysgraphia. We did not exclude subjects with additional developmental disorders that frequently occur together with dysgraphia. Our goal was to provide decision support that can be widely used to diagnose dysgraphia as a difficulty in handwriting production. The data were independently assessed by three professionals to determine whether dysgraphia was present.

**Figure 1:**
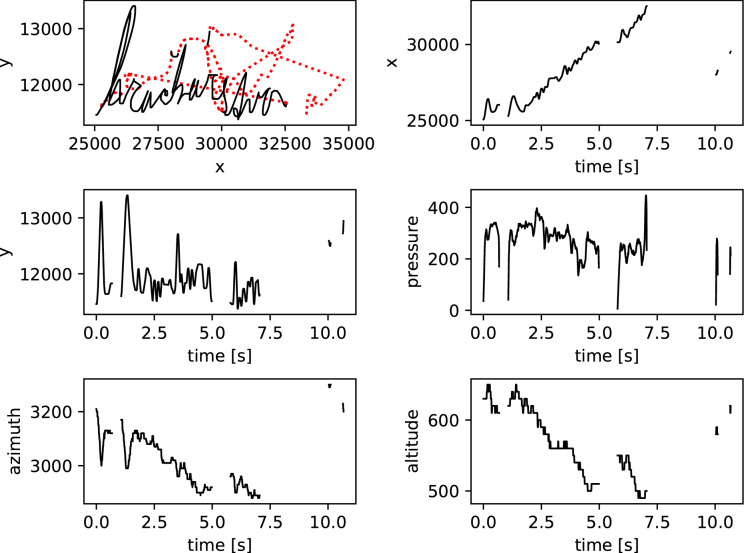
Handwriting sample of a dysgraphic child and all signals captured by the tablet. The red dotted line in the upper left figure depicts in-air movement.

Data were collected using a WACOM Intuos Pro Large tablet. The children wrote with a pen on paper that was positioned on the tablet. The tablet is capable of capturing five different signals: pen movement in the x-direction, pen movement in the y-direction, the pressure of the pen on the tablet surface, and the azimuth and altitude of the pen during handwriting. Examples of these signals as captured by the tablet are depicted in Fig. 1. Additionally, the tablet indicates whether the pen tip is touching the surface (on-surface movement) or moving above the surface (in-air movement).

### Handwriting features

To acquire the characteristics of handwriting, we extracted several handwriting features that characterize the spatiotemporal and kinematic aspects of handwriting. We focus solely on the spatiotemporal and kinematic features since these represent the gold standard of handwriting features and are frequently used to evaluate handwriting^17^. Several more advanced features, such as non-linear features and spectral features, have been proposed, but including these does not always help to increase the accuracy of the model^27^. Adding more features also increases the dimensionality of the data. High dimensionality frequently leads to overfitting of the data, which negatively impacts the prediction performance of the classification algorithm.

From a signal processing point of view, there are several groups of features, depending on how these features are extracted. Velocity, acceleration, jerk, pressure, altitude and azimuth were first extracted in the form of a vector of the same length as the handwriting sample record. To use this as an input to the machine learning algorithm, we determined the statistical properties of the vector by calculating the mean, median, standard deviation, maximum, and minimum. Since the maximum and minimum can be distorted by outliers, we decided to include the 5th percentile and 95th percentile. These selections do not take into account five percent of the most extreme values and are not as strongly influenced by abrupt peaks in the signal.

Another group of features is related to the handwriting segment, which refers to continuous movement between the transition from the in-air to on-surface states and vice versa. In some studies, this movement is denoted as stroke. However, in handwriting research, stroke is frequently understood as a kind of ballistic movement that is not necessarily equal to the segment between transitions. To avoid confusion, we henceforth use the term segment. We calculated the duration, vertical/horizontal length, height and width of a segment. Since there were multiple segments for each handwriting task, we calculated the mean, median, standard deviation, maximum, and minimum for each task. The last group of features was represented by scalar numbers; therefore, no statistical functions were calculated. These numbers included the number of pen lifts, number of changes in velocity/acceleration handwriting duration, and vertical/horizontal length. To capture the inclination of handwriting (some subjects do not place handwriting at the same height but tend to deviate from the row), we added novel features to express the difference between the minimum/median/mean/maximum y-position of the first and the last segment and the variance in the minimum/ median/mean/maximum of the segment’s y-position. In the sentence handwriting task, we extracted the majority of these features from in-air movement. Pressure, altitude, and azimuth are not recorded during in-air movement, so features related to these modalities were omitted for in-air movement.

Altogether, 133 on-surface movement features were extracted for every task, and an additional 112 in-air movement features were extracted for the sentence writing task. All extracted handwriting features are summarized in Table 2. All features were standardized on a per-feature basis to obtain zero mean and unit variance for further processing.

**Table 2:** Extracted handwriting features.

Feature	Feature description
Velocity	Overall change of position for a certain time mean, median, standard deviation, maximum, minimum, 5th percentile, 95th percentile)
Vertical/horizontal velocity	Overall change of vertical/horizontal position for a certain time (mean, median, standard deviation, maximum, minimum, 5th percentile, 95th percentile)
Acceleration	Change of speed for a certain time (mean, median, standard deviation, maximum, minimum, 5th percentile, 95th percentile)
Jerk	Rate of change of acceleration (mean, median, standard deviation, maximum, minimum, 5th percentile, 95th percentile)
Vertical/horizontal jerk	Rate of change of acceleration in vertical position (mean, median, standard deviation, maximum, minimum, 5th percentile, 95th percentile)
Pressure	The pressure of the pen tip on the surface (only for on-surface movement) (mean, median, standard deviation, maximum, minimum, 5th percentile, 95th percentile)
Altitude	The angle of the pen in the horizontal plane (only for on-surface movement) (mean, median, standard deviation, maximum, minimum, 5th percentile, 95th percentile)
Azimuth	The angle of the pen with respect to the vertical axis (only for on-surface movement) (mean, median, standard deviation, maximum, minimum, 5th percentile, 95th percentile)
Temporal duration of the segment	(Mean, median, standard deviation, maximum, minimum)
Length of the segment	(Mean, median, standard deviation, maximum, minimum)
Length in the vertical/horizontal direction	(Mean, median, standard deviation, maximum, minimum)
Width/height of the segment	(Mean, median, standard deviation, maximum, minimum)
Pen lifts	Number of pen lifts during writing
Number of Changes in velocity	Number of local extrema of velocity
Number of Changes in acceleration	Number of local extrema of acceleration
Duration	Total writing time
Length	Length of the writing movement
Vertical length	Length of the writing movement in the vertical position
Horizontal length	Length of the writing movement in the horizontal position
Diff end segments	Difference between the minimal/median/mean/max of the y-positions of the first and last segments
Diff pre-end segments	Difference between the minimal/median/mean/max of the y-position of the second and penultimate segments
Variance of y position	Variance of the min/max/median/mean of the segment’s y-position

The statistical functions applied to vector features are provided in brackets.

### Preliminary data analysis and visualization

The feature extraction stage produced 133 features per task for every data sample. Before proposing the classification model, we analysed the distribution of the data in the features space. The aim was to identify patterns that could be used in advance for the classification model.

**Figure 2:**
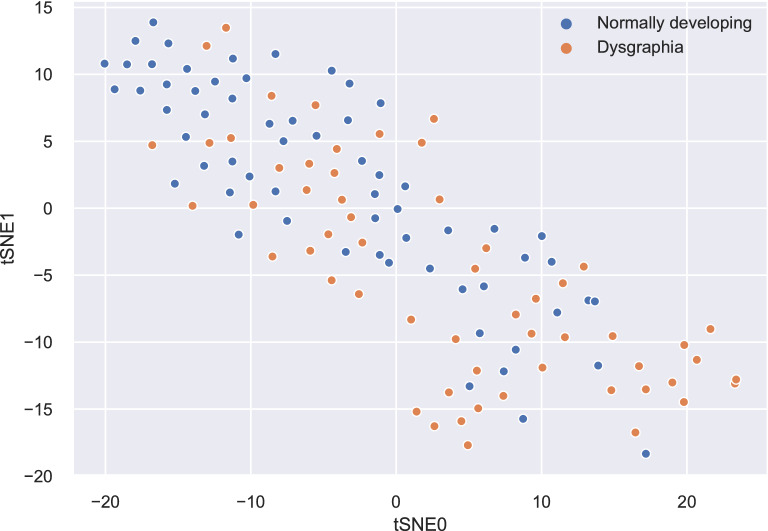
tSNE two-dimensional visualisation of the dataset.

To obtain initial insights on the data, we employed principal component analysis (PCA) and the t-distributed stochastic neighbour embedding (tSNE) method^28^ to visualize the dataset. The tSNE method is a dimensionality reduction approach frequently used in data science and machine learning to visualize high-dimensional data. The tSNE method converts high-dimensional distances between data points in Euclidean space to low-dimensional space. The dimensionality reduction is non-linear and adapts to the underlying structure of the data by performing different transformations on different regions. The two-dimensional map in Fig. 2. shows the distribution of data points representing samples of subjects with dysgraphia and normally developing subjects. Although there is some tendency of the data points representing dysgraphic subjects to accumulate in the lower right part of the space and those representing control subjects to accumulate in the upper left corner, in general, the data points are blended, indicating the following: even when using non-linear transformation, no simple rule can be derived to separate the two groups, and some samples have apparent dysgraphic characteristics, but in some cases, it is difficult to recognize these characteristics.

**Figure 3:**
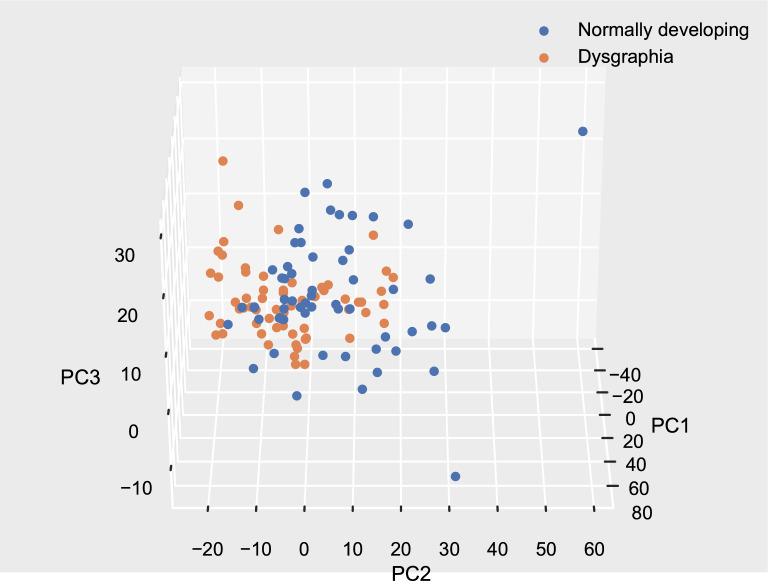
First three components of the PCA in 3D space. The total amount of variance explained by the first three components is 0.46.

Similar patterns can be found by displaying the first three principal components in three-dimensional space as depicted in Fig. 3. Similarly to the results of tSNE, there are regions in which only dysgraphic samples or only data points representing normally developing subjects are grouped, but there is a high amount of overlap of data points in space. This indicates that the linear classifier cannot separate the two classes and that a more sophisticated classifier needs to be employed.

### The most relevant features for dysgraphia detection

In the previous section, the features were compressed to two/three components to provide better visualization of the data. In this section, we analyse all original features obtained in the feature extraction stage. The feature extraction stage produces 133 features per task, and thus there are more than one thousand features in total when they are merged from all tasks. The classification model for predicting dysgraphia that will be proposed later acts as a black box. There is no clear interpretable relationship between the input features and the final decision. Therefore, before building the prediction model to detect dysgraphia, we analysed the features that are relevant for diagnosis. This analysis is important for the interpretation of the model and to gain better insight into handwriting deterioration due to dysgraphia. We employed the supervised feature selection (FS) method to identify the most relevant features. FS techniques are frequently used to reduce the dimensionality of the data by selecting only the most important features for further processing. Here, we used FS only to identify the most important features and did not reduce the dimensionality of the data for the prediction stage.

We utilized the recently proposed weighted k-nearest neighbours FS (WkNN-FS), which showed very good ability to identify relevant features^29^. The WkNN-FS identified 150 features out of 1176 as relevant for classification. The features that were evaluated by WkNN-FS as not relevant for the predicted variable have zero weight. A higher weight indicates higher importance for prediction. The weights assigned to each of the selected features are depicted in Fig. 3. The weights of the same features for the different tasks are stacked together to determine which handwriting features have the greatest weight. Since some features could not be extracted for in-air movement (as indicated in Table 2), we show these separately. The 15 features with the greatest weights, starting with the highest weight, are number of pen lifts, vertical length, maximum segment vertical length, minimum segment height, difference between the maximum y-positions of the second and penultimate segments, 5th percentile of acceleration, maximum segment length, length of writing movement, standard deviation of segment height, altitude mean, difference between the median y-positions of the first and last segments, altitude median, mean segment vertical length, standard deviation of segment vertical length, and minimum pressure. The weights of these features account for almost 50% of the total feature weights. Note that these are mostly the features that showed some relevance in multiple handwriting tasks. Among these, maximum segment vertical length, minimum segment height, difference between the maximum y-positions of the second and penultimate segments, maximum segment length, standard deviation of segment height, difference between the median y-positions of the first and last segments, mean segment vertical length, and standard deviation of segment vertical length are newly proposed features. They seem to be as important as frequently used kinematic features like speed and acceleration.

**Figure 4:**
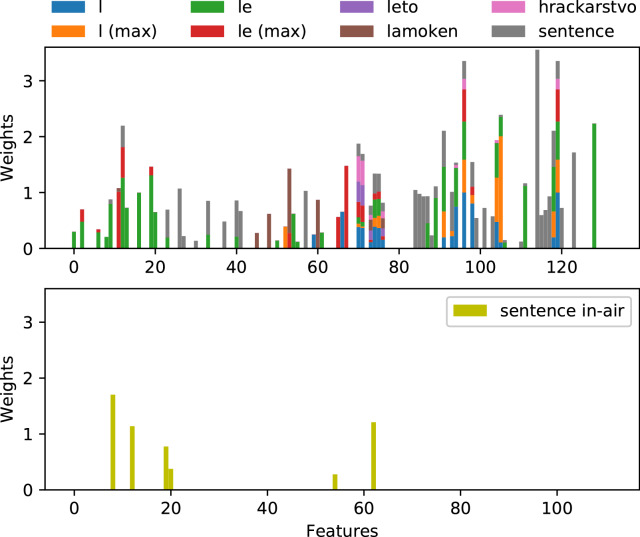
Weights assigned to handwriting features for different handwriting tasks. The upper image shows weights of features extracted from on-surface movement. The bottom image shows weights assigned to features extracted from in-air movement (sentence task only).

Some features obtained quite a high weight from only a single handwriting task, such as number of pen lifts for the sentence task (position 114 in the upper bar plot in Fig. 4) and difference between the maximum y-positions of the second and penultimate segments for task “le” (position 128 in the upper bar plot in Fig. 4). On the other hand, features such as vertical length (position 119), maximum segment vertical length (position 96), altitude mean (position 70) and altitude median (position 71) were selected as relevant in multiple handwriting tasks and yielded high weights after the weights per individual task were summed.

Only the sentence task was used to extract handwriting features from in-air movement. We also experimented with in-air features from other tasks, but these features provided no benefit for prediction performance. From the available features, only six were selected by the WkNN-FS algorithm: acceleration median, 95th percentile of horizontal jerk, 5th percentile of acceleration, 5th percentile of jerk, 95th percentile of jerk and 5th percentile of horizontal acceleration. These features showed some relevance to prediction performance.

### Classification model

To differentiate between handwriting samples from normally developing children and those from children with dysgraphia, we proposed a classification model that would learn the difference between the two groups. The model is represented by a non-linear function that takes the handwriting features as input and provides a diagnosis decision.

Our main goal was to develop a prediction model that is capable of distinguishing dysgraphic handwriting from normally developing handwriting. From a machine learning point of view, this is basically a binary classification task. For binary classification, a large number of algorithms are available, ranging from quite simple decision trees to complex deep NNs that currently pertain to many areas and provide unprecedented prediction performance. However, deep NNs require large amounts of data and therefore are not suitable for domains where it is difficult (or expensive) to obtain data. Even without deep NN methods, there are still many methods to choose from. We utilized the popular Python scikit-learn^30^ module, which implements most of the established machine learning algorithms. To find the optimal solution for our task, we experimented with several classification algorithms and employed an automated machine learning tool, TPOT^31^, that optimizes machine learning pipelines using genetic programming. For TPOT, we used a default configuration that tries a combination of different classifiers and pre-processing techniques. However, we found the best classifier by manual searching based on experience. We focused mostly on non-linear classifiers that are able to model complex non-linear patterns in data, such as ensemble classifiers and kernel classifiers. From these classifiers, the most promising performance was achieved by adaptive boosting (AdaBoost)^32^, random forest^33^ and SVM^34^. The prediction accuracies of these three methods were similar, which gave us confidence in the results.

## Results

The prediction performance of the proposed model was measured by

1 $$\begin{aligned} accuracy= & {} \frac{TP+TN}{TP+TN+FP+FN} \cdot 100 \%, \end{aligned}$$

2 $$\begin{aligned} specificity= & {} \frac{TN}{TN+FP}\cdot 100 \% \end{aligned}$$

and

3 $$\begin{aligned} sensitivity= \frac{TP}{TP+FN}\cdot 100 \%. \end{aligned}$$

Here, true positive (TP) and false positive (FP) represent the number of correctly identified dysgraphic subjects and the number of subjects diagnosed as dysgraphic but normally developing. Similarly, true negative (TN) represents the total number of correctly identified normally developing subjects, and false negative (FN) represents dysgraphic subjects evaluated as normally developing.

Classifier validation was conducted using stratified tenfold cross-validation, and the whole process was repeated ten times. Classification accuracy, sensitivity, and specificity over the ten repetitions were averaged. Training and testing features were normalized before classification on a per-feature basis to obtain zero mean and unit variance. We did not employ feature selection since this did not yield any increase in prediction performance.

To optimize performance, we tuned the hyperparameters of all classifiers. For the AdaBoost classifier, we searched the number of estimator hyper-parameters through values from 20 to 500 with step 20. For random forest, the hyperparameters were optimized by using a grid search of possible values. In brief, we searched the grid (number of estimators ($N_e$), minimum number of samples required to split an internal node ($N_s$), and number of features to consider when looking for the best split ($N_f$)) defined by the product of the set $N_e$ =[20, 40, 60 $\ldots$, 500], $N_s$ = [2, 4, 6, 8] and $N_f$ = [5, 10, 20, 30, 40]. In the case of SVM, the search space was defined by parameters C = [2e−9, 2e−7, $\ldots$, 2e7, 2e9] and gamma = [2e−9, 2e−7, $\ldots$, 2e7, 2e9]. The optimal hyperparameters were 340 estimators for AdaBoost; $N_e$ =60, $N_s$ = 4 and $N_f$ = 5 for random forest classifier; and C = 4 and gamma = 2e−9 for SVM.

The prediction performance results in terms of accuracy, sensitivity and specificity are shown in Table 3. The best performance was achieved by the AdaBoost classifier, with 79.5% prediction accuracy. Competitive performance was provided by SVM and random forest classifier, which lagged by only a few percentage points. The classification accuracy of other evaluated classifiers, such as naive Bayes, decision trees, k-nearest neighbours and logistic regression, was notably lower; therefore, we do not report these results here.

We show the classification accuracy for different handwriting tasks and the classification accuracy when all tasks are used together. Notably, the performance of all reported classifiers was quite similar when all tasks were merged but varied when single handwriting tasks were compared. For the AdaBoost classifier, the task contributing most to prediction accuracy was “hračkárstvo”, yielding 76.2% prediction accuracy, only slightly less than the 79.5% accuracy obtained by utilizing all handwriting tasks. Writing the word “hračkárstvo” is quite difficult, so we assume that it requires more skills and higher cognitive load, which can make the manifestation of dysgraphia more apparent. The accuracy scores of the other two models were also quite high, 72.5% for SVM and 72.3% for RF classifier. On the other hand, the model based on the letter l and syllable le written at maximal speed appeared to have lower prediction accuracy for all classifiers. Interestingly, using tasks *leto* and *lamoken* to train the model resulted in the highest accuracies for SVM and RF classifier but an accuracy of only approximately 66% for AdaBoost.

**Table 3:** Prediction accuracy, specificity and sensitivity of three classification models SVM, AdaBoost and RF for different handwriting tasks.

Task		AdaBoost	SVM	RF
l	Acc	$68.2\pm 2$	$73.1\pm 1$	$75.9\pm 2$
	Spe/Sen	$64.5\pm 4/55.8\pm 4$	$75.8\pm 4/70.5\pm 4$	$77.6\pm 2/74.3\pm 4$
l (max)	Acc	$60.4\pm 3$	$63.4\pm 2$	$67.4\pm 1$
	Spe/Sen	$64.6\pm 4/55.9\pm 4$	$79.1\pm 2/46.2\pm 9$	$73.3\pm 3/61.6\pm 4$
le	Acc	$67.6\pm 2$	$67.8\pm 2$	$71.6\pm 1$
	Spe/Sen	$73.5\pm 4/61.4\pm 3$	$76\pm 2/58.7\pm 3$	$78.1\pm 3/64.5\pm 4$
le (max)	Acc	$61\pm 3$	$66.1\pm 1$	$66.2\pm 2$
	Spe/Sen	$65.6\pm 4/55.6\pm 3$	$78.3\pm 6/52.8\pm 7$	$75.5\pm 3/62.3\pm 3$
Leto	Acc	$65.8\pm 2$	$73.4\pm 1$	$76.2\pm 1$
	Spe/Sen	$69.5\pm 5/61.5\pm 4$	$83.2\pm 3/62.6\pm 4$	$83.7\pm 1/68.2\pm 2$
Lamoken	Acc	$66.4\pm 3$	$76.4\pm 1$	$74.3\pm 2$
	Spe/Sen	$73.3\pm 5/58.6\pm 4$	$85.1\pm 4/66.8\pm 5$	$84.8\pm 2/62.7\pm 3$
Hrackarstvo	Acc	$76\pm 2$	$72.5\pm 1$	$72.3\pm 1$
	Spe/Sen	$79.5\pm 3/73.1\pm 3$	$60.7\pm 5/63.8\pm 5$	$77.9\pm 4/66.1\pm 5$
Sentence (on-surface)	Acc	$61.8\pm 3$	$72.6\pm 2$	$71.7\pm 2$
	Spe/Sen	$62.4\pm 3/61.2\pm 5$	$85.9\pm 3/58.1\pm 1$	$77.3\pm 3/65.6\pm 3$
Sentence (on-surface + in-air)	Acc	$64.4\pm 2$	$69.7\pm 1$	$70.9\pm 1$
	Spe/Sen	$66.1\pm 3/62.9\pm 2$	$88.5\pm 2/48.9\pm 2$	$76.5\pm 2/64.6\pm 3$
All	Acc	$79.5\pm 3$	$78.8\pm 2$	$77.6\pm 1$
	Spe/Sen	$76.7\pm 2/79.7\pm 5$	$82.4\pm 4/74.5\pm 4$	$83.3\pm 2/71.4\pm 3$

## Discussion

In this study, we use a set of machine learning techniques to distinguish between dysgraphic and non-dysgraphic children. Compared with more traditional clinical analyses that focus mostly on static features and may be prone to subjective bias of the examiners, data-driven methods have the potential to help professionals diagnose disorders in children more objectively in the future^9^. Using customised tablets, researchers and clinicians alike can gather more data, which can be used to search for patterns that are not apparent when using traditional pen-and-paper tests. New methods of data acquisition and data analysis may even lead to the identification of various subtypes of dysgraphia and allow for more effective treatments^35^.

Our results show it is possible to discriminate between dysgraphic and non-dysgraphic children with $79.5\%$ accuracy on a sample of children of different ages using the AdaBoost algorithm and to a similar extent using the RF and SVM algorithms. AdaBoost is representative of ensemble classifiers, which outperform other types on classifiers on many real-world classification tasks^36^.

Several features seemed to be relevant for discriminating dysgraphic and non-dysgraphic children. In line with other studies^16^, pressure and pen lifts were among the features with high discriminatory potential. In our study, we identified three types of relevant features: static features, kinematic and dynamic features and other features. Several previous studies, such as Mekyska^18^ and Asselborn^9^, utilize the same types of features. Although the above-mentioned studies report similar sets of features, most of the individual features do not overlap across studies. We speculate that features in different groups intercorrelate and that rather than one or two strong features, there might be a cluster of intercorrelated features that provide a more accurate account of the disorder. However, the respective weights of features in different clusters may vary from sample to sample.

In our analysis, we identified a smaller subset of features that were selected by WkNN-FS as the most relevant for the predicted variable. The 150 selected features constitute only 12% of all extracted features, so many features are not relevant for diagnosis. However, when using the reduced subset of features for classification, the prediction accuracy slightly decreased. Even though this may seem surprising, it is a known phenomenon. The relevance of features does not imply optimality (optimality in the sense that the accuracy of the induced classifier is maximal)^37^. Therefore, we decided to use the complete feature set for classification since this yielded the highest accuracy score.

Our study makes several contributions to the literature on dysgraphia and machine learning.

First, this study provides new data and insights on automatic testing of dysgraphia and confirms that machine learning approaches are promising tools for objective diagnosis. It provides new data that can be used to test machine algorithms, and upon request, we will share the features extracted from our data for use by other researchers. More data will enable better validation of machine learning approaches for dysgraphia detection. When algorithms prove efficient on a broad spectrum of samples, they will be more robust and useful in clinical practice. Automatic testing would allow for more efficient screening of the population and bring enormous benefits to children who would otherwise remain undiagnosed. Early intervention based on screening would increase their academic potential, reduce stress levels and boost self-esteem.

Second, we acquired the data using a new orthography (Slovak), thus expanding the list of orthographies used in research thus far beyond Hebrew^18,19^, French^9^, Indonesian^20^ and English^38^. New orthographies are important for determining whether the algorithms used are generalisable for different cultures or whether orthography-specific algorithms should be developed for dysgraphia identification.

Third, our methodology was developed in order to improve over methodologies used in other studies in the field. In particular, we aimed to avoid the critique of Asselborn’s research^9^ by Deschamps^39^ by using the same tablet for the whole sample and having all subjects tested for dysgraphia. In addition, compared with the study by Mekyska^18^, who included only pupils in the third grade who used their right hand as the dominant hand, our sample was more diverse, which may also explain the lower accuracy of our results.

As with other studies, one drawback of our study is the limited sample size. With a limited number of samples, it is challenging to train the machine learning algorithms to achieve optimum performance. This may explain the differences in results produced by different machine learning algorithms.

The features relevant to discrimination are not always consistent among studies. The reasons for this inconsistency are not clear but may include small sample datasets, different kinds of dysgraphia that are not accounted for, and different manifestations of dysgraphia in different age groups, among other factors. We expect that further research will provide insights on the question of how orthography-sensitive machine learning methods are. New research suggests^35^ that different features may be more predictive for different subtypes of dysgraphia. We speculate that different features could also be more predictive in different developmental stages. It also appears that the machine learning approach to diagnosing dysgraphia is task-sensitive. In our study, writing more demanding words (hračkárstvo, lamoken) seemed to invoke more important features than writing simpler words. This finding suggests that there may be room to devise specific handwriting tasks for dysgraphia that are better suited to machine evaluation than the traditional tests developed for human testers.

How dysgraphia originates and which brain centres are involved in this and similar disorders are still not completely clear. The handwriting process is a complex task involving perception, motor skills and memory^40^ and possibly motivation^9^ and coordination of all of the above, and we can pinpoint some of the brain centres that are involved in the process. However, we know little about how the parameters of brain function correlate with the severity of dysgraphia. Machine learning methods evaluate the outcome of the writing process but do not directly analyse memory and perception. They test features of motor behaviour, such as pressure, acceleration, and number of pen lifts. We may speculate that the handwriting process is an interaction of three NNs: the network for perception recognizes the text, the network for long- and short-term memory interacts with the perceptive network to find appropriate motor equivalents, and the motor network realizes the outcome on the paper. Dysgraphia may result from the malfunctioning of any or all of these networks or from errors in their coordination. As neuroscientific research on dysgraphia and related disorders achieves new results, it will become possible to better focus prediction methods. New data may provide new insights on how dysgraphia develops, whether sex differences exist and how age correlates with different aspects of dysgraphia.

## Conclusions

Our study provides new data, a new orthography and an algorithm not previously used for dysgraphia recognition. We introduced several new features that have not previously been used to evaluate handwriting and dysgraphia. These features proved relevant for diagnosis and, moreover, offer a high level of interpretability. Features such as maximum segment vertical length, minimum segment height, and difference between maximum y-positions of the second and penultimate segments can be directly related to changes in handwriting due to dysgraphia. In conclusion, the proposed approach was able to recognize dysgraphic handwriting with almost 80% accuracy; however, the dataset includes subjects aged 8–15 years. This is a relatively wide age range, especially for handwriting, since handwriting is still developing and changing during these years. This makes classification tasks more challenging than in more focused datasets.

The proposed model can be employed as part of a decision support system to assist professionals in occupational therapy to provide more objective diagnosis. Some commercially available conventional tablets now offer the possibility of capturing handwriting, which would allow a whole decision support system to be implemented on a tablet device at relatively low cost, thus opening possibilities for extensive screening of children for dysgraphia in schools.

The limitations of our study lie in the fact that we used only Slovak orthography and tested children in a relatively broad age range with fewer cases in separate age groups, so we could not pinpoint differences between children of different ages. Additional studies are necessary to identify whether the features proposed by us and others^9,18^ are valid for other orthographies and other age cohorts.

## Supplementary information

Supplementary Information.
