# Supplementary material for: Dysgraphia detection through machine learning
Source: Sci Rep. 2020 Dec 9;10:21541. doi: 10.1038/s41598-020-78611-9 (PMC7725992; doi:10.1038/s41598-020-78611-9)
Supplement: Supplementary file 1 — Supplementary Information. [file 41598_2020_78611_MOESM1_ESM.pdf]

Dysgraphia detection through machine learning

Peter Drotar[1] **and Marek Dobeš**[2,*]

[1]Department of Computers and Informatics, Technical University of Košice, 04001 Košice, Slovakia
[2]Centre for Social and Psychological Sciences, Slovak Academy of Sciences, 04001 Košice, Slovakia
[*]dobes@saske.sk

Template used for handwriting acquisition:

Meno, priezvisko:	Dátum vyšetrenia:	Ročník ZŠ:	Vek:	Examin.:

l

l

le

le

leto

lamoken

hračkárstvo

V lete bude teplo a sucho

ID hodnotitela:<br>hodnotenie:	ID hodnotitela:<br>hodnotenie:	ID hodnotitela:<br>hodnotenie:
